# SensAA—Design and Verification of a Cloud-Based Wearable Biomechanical Data Acquisition System

**DOI:** 10.3390/s24082405

**Published:** 2024-04-09

**Authors:** Jonas Paul David, David Schick, Lorenz Rapp, Johannes Schick, Markus Glaser

**Affiliations:** Zentrum für Zuverlässige Mechatronische Systeme (ZMS), Aalen University, 73430 Aalen, Germany

**Keywords:** medical engineering, biomechanics, exoskeleton, active knee orthosis, motion analysis, sensor technologies, human activity recognition, wearable sensors

## Abstract

Exoskeletons designed to assist patients with activities of daily living are becoming increasingly popular, but still are subject to research. In order to gather requirements for the design of such systems, long-term gait observation of the patients over the course of multiple days in an environment of daily living are required. In this paper a wearable all-in-one data acquisition system for collecting and storing biomechanical data in everyday life is proposed. The system is designed to be cost efficient and easy to use, using off-the-shelf components and a cloud server system for centralized data storage. The measurement accuracy of the system was verified, by measuring the angle of the human knee joint at walking speeds between 3 and 12 km/h in reference to an optical motion analysis system. The acquired data were uploaded to a cloud database via a smartphone application. Verification results showed that the proposed toolchain works as desired. The system reached an RMSE from 2.9° to 8°, which is below that of most comparable systems. The system provides a powerful, scalable platform for collecting and processing biomechanical data, which can help to automize the generation of an extensive database for human kinematics.

## 1. Introduction

The most common activities of daily living (ADL) are walking and climbing stairs [1]. The ability to perform ADLs with ease is essential for the quality of life of patients or elderly persons. This is especially important, as disorders of the human gait pattern increase significantly with age. A study by Mahlknecht et al. [2] revealed that the prevalence of gait and balance disorders ranges around 10% for individuals aged between 60 and 69 years and exceeds 60% for those aged over 80 years. In the context of an ageing society, interest in effective and affordable treatment methods for gait disorders is increasing. There are multiple approaches to reduce the impairment of gait disorders. Among them are orthoses and exoskeletons, which can be used to support or even regain lower extremity functions [3,4,5,6,7,8,9,10,11]. To develop and validate such devices and to adapt them to the user, long-term gait observation is necessary. Parameters such as knee joint torque and angle, hip joint angular velocity, and the speed of the subject’s movement can be derived based on biomechanical measurements [5]. By evaluation of these parameters over the course of a day while performing ADL, load collectives can be generated for the lower limb. The collected data are instrumental in refining the design, verification, and control of exoskeletons.

Biomechanical data can be captured with motion analysis systems (MASs). There are several methods for this purpose, which differ primarily in the choice of sensors.

A frequently selected method of recording motion kinetics is the use of optical systems [12]. An example of such a system has been described by Riener et al. [13].

This methodology with motion-detecting cameras has been proven for decades in biokinematics but is inappropriate for a wearable biomechanical analysis system. Optical systems can record human motion very accurately but are cost-intensive and restrict the recording spatially into a stationary environment equipped with an array of cameras [12,14]. However, the precise data and the large amount of test results in the literature can be used for the verification of a new measurement system [13,15].

Wearable systems based on inertial measurement units (IMUs) have been recently a subject of extensive research [12,14,16,17,18,19,20,21,22,23,24,25,26,27,28,29,30,31,32,33,34,35,36,37,38,39,40,41,42,43]. These systems, while not yet delivering the same accuracy as optical systems, can be operated everywhere and not only in a stationary environment equipped with cameras. Commercial solutions for inertial motion capturing have existed since the early 2000s. In 2008, Cloete et al. [42] benchmarked the commercially available MOVEN motion capture system by XSENS, comparing it to a clinically used optical system. They concluded that the system can measure the inclinations of body parts and joint angles with reasonable accuracy, while being exceptionally fast to set up and flexible in its use [42]. However, the tested commercial solutions are still expensive, with the latest version of the XSENS motion capture suit costing more than USD 4000.

Due to their advantages, IMU sensors are the most commonly used wearable technology for gait analysis [39]. But this technology is still not used to its full potential. Benson et al. reviewed over 230 MASs for running gait analysis based on IMU sensors. Twenty-eight percent of the studies were conducted outdoors, and in 33% of all studies, the analyzed distance was greater than one step or stride or 200 m [44], which means most of the systems are not designed to run long-term measurements out of the laboratory. Furthermore only 10% of the reviewed devices are used for measuring joint angles or range of motion. Benson et al. suggest shifting gait analysis research from controlled laboratory settings to more real-world environments [44].

This leads to the conclusion that there is a research gap in developing a low-cost solution for measurements of biomechanical data over the course of multiple days in an environment of daily living.

Long-term measurements are required to obtain a complete set of biomechanical data in real-life conditions. Field measurements offer a significantly larger and more realistic variety of load scenarios compared to simulating selected walking activities in a laboratory. Additionally, simulating tasks can lead to systematic errors. These errors can be avoided by measuring under real conditions.

Load collectives of the human leg will be calculated from the data of long-term measurements obtained by the proposed system. The data sets will also help to identify different walking activities (e.g., level walking, stair ascending and descending) performed by the user. This information will be used in future work for the control of an active exoskeleton for the human knee.

For this purpose, SensAA is focused on measuring the kinematics of the lower human limbs. SensAA will be used by elderly people; therefore, the use of the measurement system shall be as intuitive as possible. The wearer does not need technical knowledge to operate the system and can don and doff it on their own. No maintenance like recharging, sensor adjustment and calibration are required during runtime. The system is implemented using off-the-shelf hardware and open-source software. This reduces cost and improves accessibility of the system.

SensAA is used in activities of daily living both indoors and outdoors. It is also used all day without taking it off. Therefore, a small, compact, and wireless design is suggested, which is non-obstructive to its wearer and does not cause discomfort.

To take full advantage of this wearable design, cloud-based software for data acquisition and analysis is proposed. While the measurements are running, the data are automatically uploaded to a database on a cloud server. The server allows for central data storage and online data collection for multiple users simultaneously.

In the literature, there already are reports of approaches that use IMU sensors to measure lower limb kinematics [41,45]. However, to our knowledge, no device has yet been described that combines all desired goals. These approaches lack at least one of the following aspects:

- Low-cost solution [28,36,37,40]
- Easy setup and low calibration effort [25,27,28]
- Compact and wireless design [14,34,46]
- Continuous and secure data upload to cloud server [26,28,34,36,37,40,46,47,48]
- Data upload outdoors without WIFI connection [28,36]
- Energy optimization for long-term measurements [28,36,37,40]
- Secure data transfer for multiple users [26,34,37,40,46,47,48]

This paper presents a novel all-in-one solution that combines solutions for accuracy, usability, and security to close the shown research gap.

## 2. Materials and Methods

### 2.1. System Architecture

The system SensAA consists of the following three main components:

- Wearable Sensor Boxes
- Android Smartphone Application
- Cloud Server System

**Figure 1:**
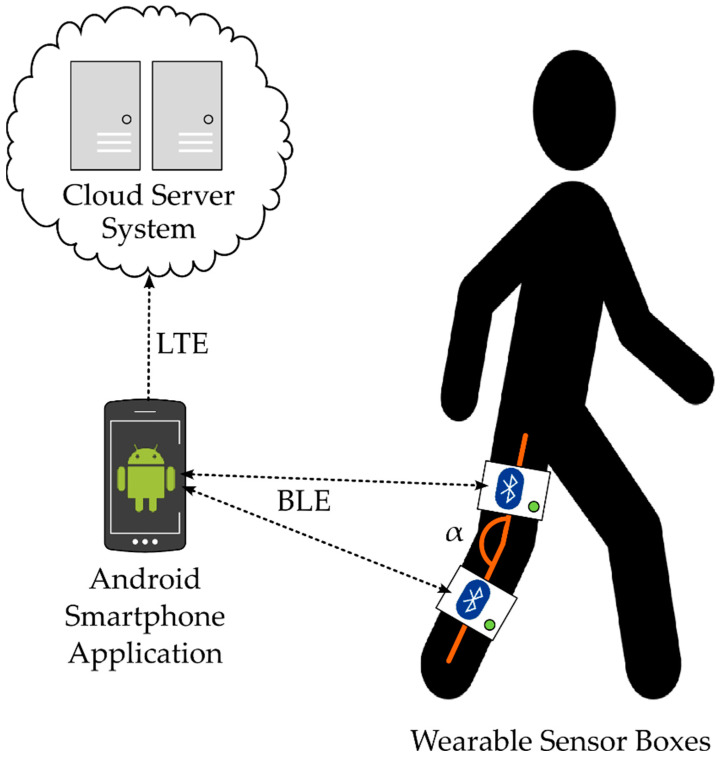
System overview of SensAA.

The components and their interfaces are illustrated in Figure 1.

SensAA uses IMU sensors to measure joint angles. This sensor principle enables the measuring system to be used independently in every environment of daily living.

The IMU sensors measure acceleration and angular velocity to observe their inclination relative to the gravity vector on Earth. At least two sensors are required to measure the angle at one joint axis. Each sensor is integrated in a housing, a wearable Sensor Box, which also contains a battery and a Bluetooth^®^ Low Energy (BLE) module (see Section 2.2).

In order to measure the angle of the human knee joint, one Sensor Box is attached to the thigh and another one to the shank of a leg. The sensors are placed parallel to the sagittal plane of the user’s body.

The data collected are transmitted wirelessly via BLE from the sensors to a smartphone (see Section 2.3). The smartphone runs an Android application, which receives the data via BLE notifications and forwards them to a Cloud Server System over the cellular network. The Cloud Server provides a read and write interface to a central database for up- and downloading datapoints (see Section 2.4). The link to this database gives the possibility to create data backups regularly. Since the server is accessible from a public address, appropriate measures must be taken to ensure the authenticity and authority of the users.

**Figure 2:**
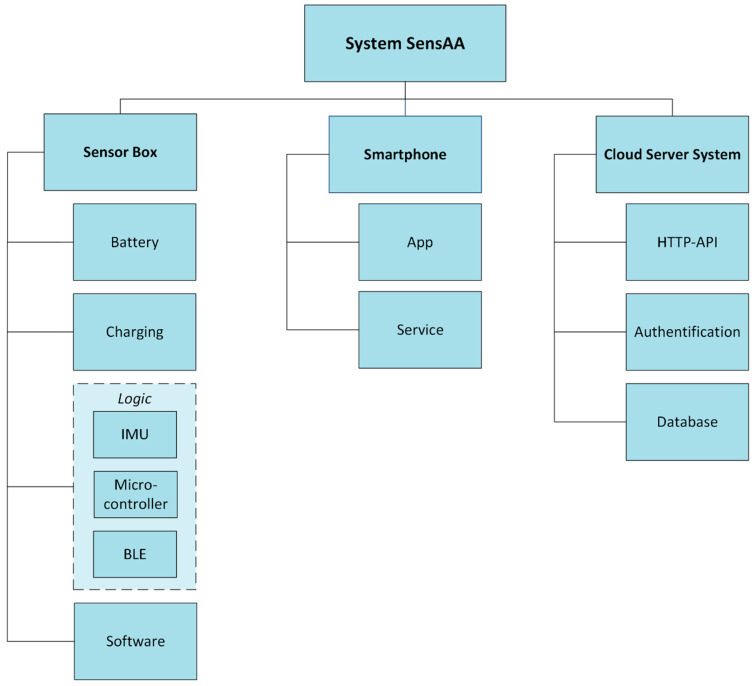
System architecture of SensAA.

Figure 2 shows the system architecture of SensAA. The three main components and their sub-architectures are described in Section 2.2, Section 2.3 and Section 2.4.

### 2.2. Sensor Box

#### 2.2.1. Electronic Structure

**Figure 3:**
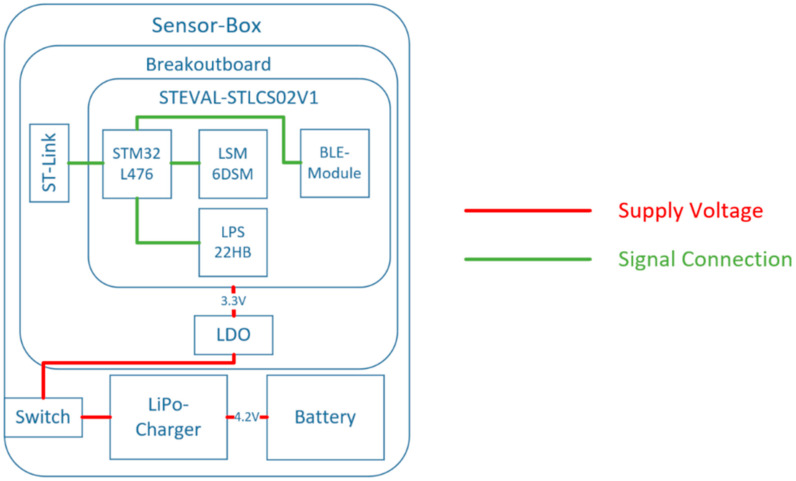
Electronic structure of SensAA.

The Sensor Boxes with integrated inertial sensor and microcontroller are designed to have a small form factor while maintaining a long battery life. Figure 3 shows a block diagram of the electronic structure and interfaces of SensAA.

The long duration of recordings requires the use of energy-efficient hardware and an appropriately dimensioned power source. IMU sensors based on Micro-Electro-Mechanical Systems (MEMS) technology have negligible power consumption and are well suited for the proposed application. Processors and wireless transmitters also have to be carefully selected to keep the power consumption to a minimum. For this reason, Bluetooth^®^ Low Energy is the preferred protocol for transmitting data wirelessly to a smartphone (see Section 2.3).

The compact STEVAL-STLCS02V1 (STEVAL-Board) sensor board from STMicrolectronics (STMicroelectronics, Geneva—Plan-Les-Ouates, Switzerland) has been chosen. This board is inexpensive and offers a suitable interface for programming the integrated STM32-L476 microcontroller (STMicroelectronics, Geneva—Plan-Les-Ouates, Switzerland) [49]. Furthermore, its small size (13.5 mm × 13.5 mm) makes the board ideal for the intended application. The board has a built-in IMU sensor, LSM6DSM (STMicroelectronics, Geneva—Plan-Les-Ouates, Switzerland), which incorporates an accelerometer and a gyroscope. Linear acceleration can be measured up to ±16 g (gravity of the Earth) and the angular velocity up to ±2000 degrees per second (dps) [50]. BLE communication is provided by the modules integrated on the STEVAL-Board.

A 500 mAh lithium polymer (LiPo) battery (Adafruit^®^, New York, NY, USA) provides the power supply, which can be switched on and off via a slide switch. The nominal battery voltage is 4.2 V. To recharge the battery and provide long operating times, a LiPo battery charger from Adafruit^®^ (New York, NY, USA) is used [51]. This charger can be connected through a micro-USB (Universal Serial Bus).

For the connection to the supply voltage and the interface ports of the STEVAL-Board, a breakout board is required. Therefore, a custom printed circuit board (PCB) has been designed. The supply voltage of this breakout board PCB is 3.3 V, which is constantly provided by a low-dropout voltage regulator (LDO).

#### 2.2.2. Mechanical Structure

The electronic components are mounted in a 3D-printed housing, the Sensor Box. The bottom half of the housing provides cut-outs and guides for components and cables. One corner of the custom PCB is cut at an angle, which prevents the PCB from being inserted incorrectly in the housing. The PCB has an overall size of 17.5 mm × 20 mm. The housing also provides openings for the slide switch and the USB connector of the LiPo charger.

The top half of the housing includes support structures to ensure that the components do not move during measurements. While the Sensor Box is closed, these support structures are in contact with the components and hold them down securely.

**Figure 4:**
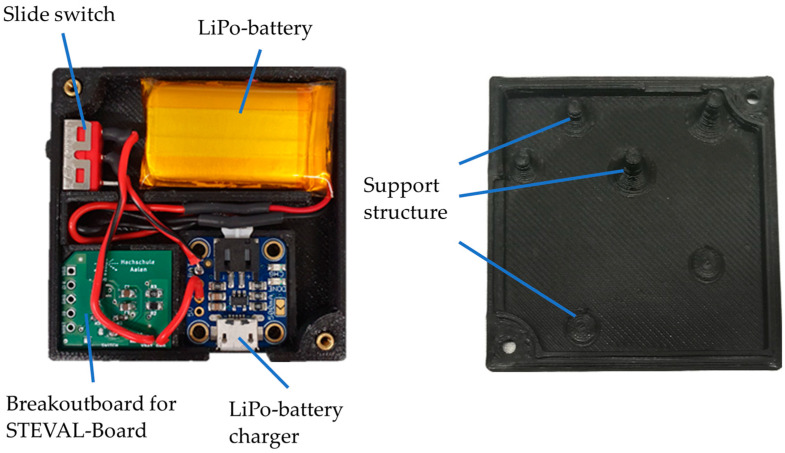
The 3D-printed Sensor Box; left: bottom half, right: top half.

The Sensor Box has the final dimensions of 53.2 mm in length, 52.5 mm in width, and 11.6 mm in height. Figure 4 shows the 3D-printed housing with the fitted components.

### 2.3. Sensor Firmware and Smartphone Application

The firmware of the sensors is implemented in the C language with the following main functions:

- Reading values from the built-in accelerometer and gyroscope
- Observing the inclination of the sensors by fusing the accelerometer and gyroscope data
- Transmission of measurement data to the Android application

The Android application provides a user interface to interact with the sensors. The application allows users to connect, disconnect, and calibrate the sensors. Communication between the application and the sensors is implemented using the BLE Generic Attribute Profile (GATT) protocol. All interactions are mapped to GATT services and characteristics. A service is a background process that may be executed even when the device display is off. The service is used to silently collect sensor data via BLE without needing any interaction with the user.

The following GATT services are provided:

- The “Metric” service provides the sensor data. For each sensor data type (acceleration, angular velocity, inclination), a GATT characteristic is defined and can be subscribed by the smartphone app. Furthermore, a GATT descriptor is added to each characteristic, describing the scale and unit of the provided metrics. Using the descriptor, the full scale of the device can be reconfigured, without reprogramming the scaling in the smartphone application.
- The “Version” service provides the firmware version running on the sensor. It features two characteristics, one for reading the major and minor version numbers and one for the exact build revision.
- The “Control” service is used to imperatively trigger different processes in the sensor firmware. Each characteristic of the service is assigned to an action in the sensor firmware. For now, it is only used to initiate the self-calibration function.

**Figure 5:**
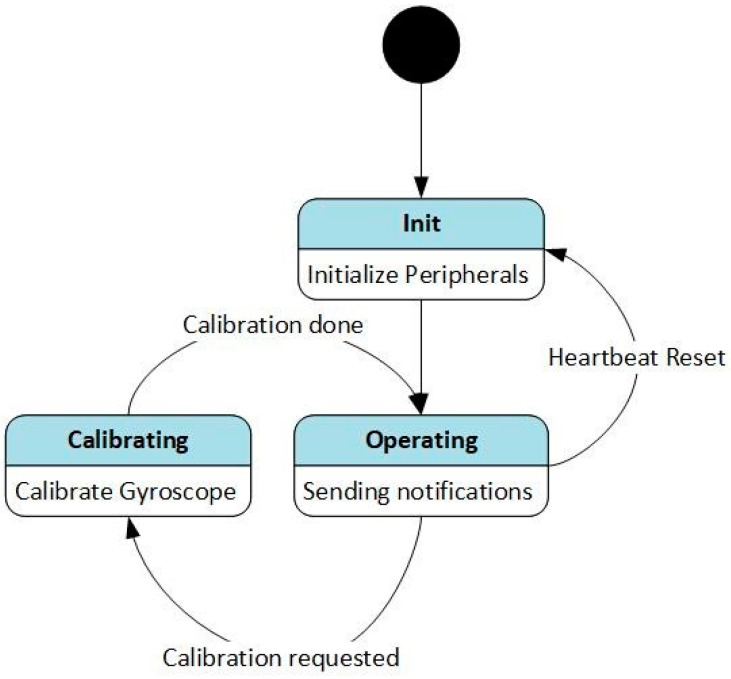
Sensor state machine of the sensor firmware of SensAA.

Figure 5 illustrates the main state machine of the sensor firmware. On reset, the system goes into the Init state. In the Init state, the microcontroller and its peripherals are configured. After initialization, the system goes into the Operating state. In the Operating state, the sensors are read, and the notifications are updated. The user can trigger self-calibration of the sensors via the “Control” GATT service. During self-calibration, the offset in the gyroscope values is calculated while the sensor is in a stationary state. This offset is then stored in flash and applied to all subsequent sensor readings. When calibration is finished, the system goes back into the Operating state, resuming characteristic updates. During operation, the sensors calculate their orientation using an extended Kalman filter (EKF)-based observer algorithm. The observer estimates the direction of the gravity vector, from which the inclination of the sensor is derived. The inclination is published via the ‘Inclination’ GATT characteristic in radians as a 32-bit floating point number.

Depending on the number of sensors connected, the sensors reach a sampling rate from at least 20 Hz (with four sensors) up to 50 Hz (with two sensors). The average frequency of walking is between 1.5 Hz and 2.5 Hz [52] and can go up to 4.5 Hz while climbing stairs [53]. According to the Nyquist–Shannon Sampling Theorem, a sampling rate of at least 9 Hz is needed to record the gait pattern of a subject correctly. Therefore, the sampling rate of SensAA is sufficient for human motion analysis.

### 2.4. Cloud Server System

The Cloud Server System has the responsibility of persistent data storage, while keeping the data accessible, integrous, and consistent. The consistency and integrity requirement can be met by using a database management system and a well-defined data model. For this purpose, a MySQL (v8.3.0) database management system is used. To enable cross-platform access to a database system, an interface application based on web technologies has been chosen. Therefore, the system communicates using the Hypertext Transfer Protocol (HTTP), which is the standard protocol for web applications. To achieve high accessibility, an architecture was developed that allows for fast deployment and updatability. The clients interacting with the Cloud Server System are connected via public, mobile networks. The Cloud Server System listens on a public IP address. When exposing the application to the internet, data security becomes an immediate concern. To prevent unauthorized access to the system all, communications are encrypted, and an authentication and authorization system is used.

**Figure 6:**
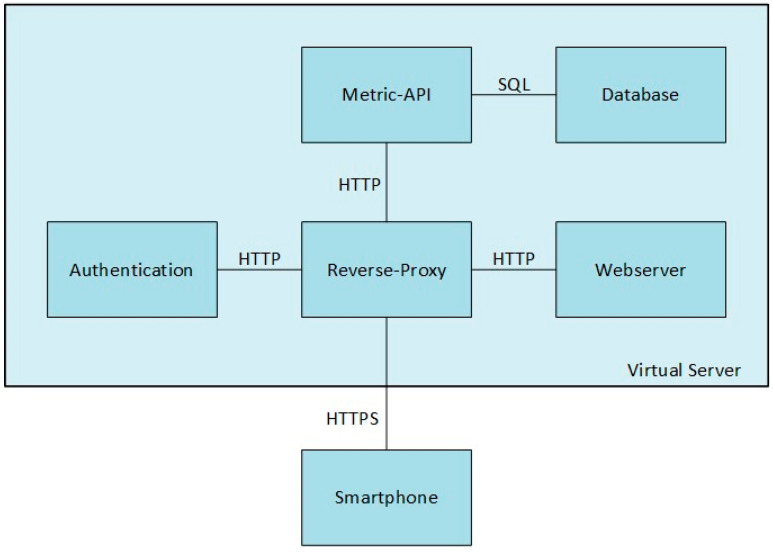
Architecture of the SensAA Cloud Server System.

Figure 6 shows the proposed Cloud Server System architecture. For simple interaction, the system acts as a single HTTP server towards the client. Internally, the system is implemented as a docker container network. Each container runs specific services, each responsible for enabling distinct functions of the system. All containers can be maintained and updated individually. This allows incremental updates, without the need to shut down the entire system. All services are connected to the clients via HTTP-based application programming interfaces (APIs). However, from a client’s point of view, it is easier to communicate with a single server offering all of these services. The API Gateway is a reverse proxy that addresses this issue. Depending on the path of the request, the request is relayed to the service handling it. To ensure data security, the API Gateway uses Transport Layer Security (TLS). The API Gateway decrypts incoming HTTP messages and relays each unencrypted message to its destination service. In this way, while the services are not handling security themselves, the API Gateway ensures that no unencrypted data can leave the system.

The API Server container hosts the Metrics API, which is responsible for accessing the database. The Metrics API provides one endpoint for each type of measurement, each reachable on a distinct HTTP path. There is a corresponding database table for each endpoint. The scheme of this table describes the measurement datatype. When inserting a datapoint, the body of the client’s request contains a Java Script Object Notation (JSON) document, which contains an array of JSON objects, each representing a single measurement. When retrieving datapoints, the body of the HTTP response contains the datapoints in the same format. To support selective retrieval of the datapoints, the endpoints provide filter options for each column of the datatype in question. The supported filter operators depend on the column datatype. A text value can only be compared for equality. For numerical types, the column can be filtered using a comparison value, with either an equality, greater than, or less than operator applied. To filter a range of values, both a greater than and a smaller than operator can be applied to the same column. The filters are passed to the API with the HTTP request query string.

### 2.5. Verification

To verify the accuracy and the data transmission reliability of the proposed inertial measurement system, several tests are carried out, which ensure that the set requirements are met and that correct measurement results are achieved. Verification includes a battery life test, a pendulum test, and a treadmill Test.

#### 2.5.1. Battery Life Test

The goal of the battery life test is to verify whether the battery-based power supply of the Sensor Boxes is sufficient for long-term recording. Sensor Boxes need to be able to record and transmit data over a complete day, at least 16 h (cf. Section 1). For this purpose, two sensor modules are switched on and connected to a smartphone via the Bluetooth^®^ Low Energy interface. This starts the data acquisition and the test. The smartphone is logged into the Cloud Server System and continuously delivers measurement data. Sensors remained stationary during the test. The sensor board has a supply voltage of 3.3 V. When the voltage drops below this threshold, the sensor is turned off, and the recording stops. This timepoint can be determined precisely because the data are recorded with absolute timestamps. The battery charge of the smartphone (in %) and the sensors (in V) are recorded regularly in one-hour intervals.

#### 2.5.2. Pendulum Test

**Figure 7:**
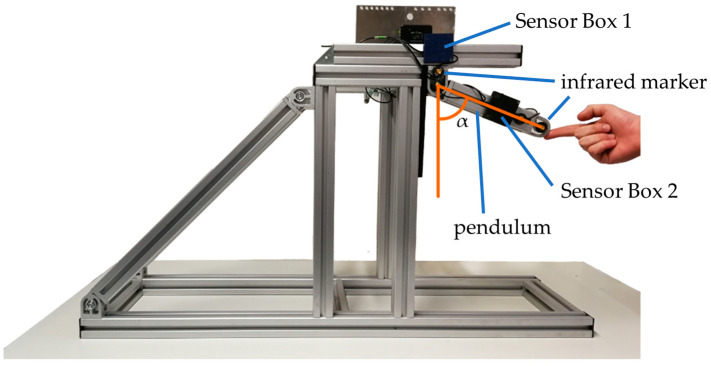
Pendulum test setup with marked angle α and two Sensor Boxes. Sensor Box 1 is fixed on a stationary vertical axis, and Sensor Box 2 is mounted on a free-swinging pendulum. Both the axis and pendulum are marked with infrared markers to measure α with an optical MAS simultaneously.

The goal of this test is to verify the EKF-based observer algorithm and to determine the accuracy of SensAA (see Section 2.3). Two wearable Sensor Boxes are attached to the setup shown in Figure 7. One Sensor Box is fixed along the vertical axis, while the other is mounted on a free-swinging pendulum. The angle α in the figure denotes the angle measured by the system. For this experiment, an optical MAS (LaiTronic GmbH, Innsbruck, Tirol, Austria, former Steinbichler) is used as reference for comparing the measurement data (cf. Section 1). The infrared markers are attached to the vertical axis and the far end of the pendulum. Therefore, the MAS measures the same angle as the wearable sensors. The sampling rate of the MAS must be configured for the test setup. The maximum sampling rate of the system is limited by the distance to the markers. Here, the MAS is set about 3 m away from the test setup. In this configuration, a sampling rate of 220 Hz resulted in clean measurements of α.

#### 2.5.3. Treadmill Test

**Figure 8:**
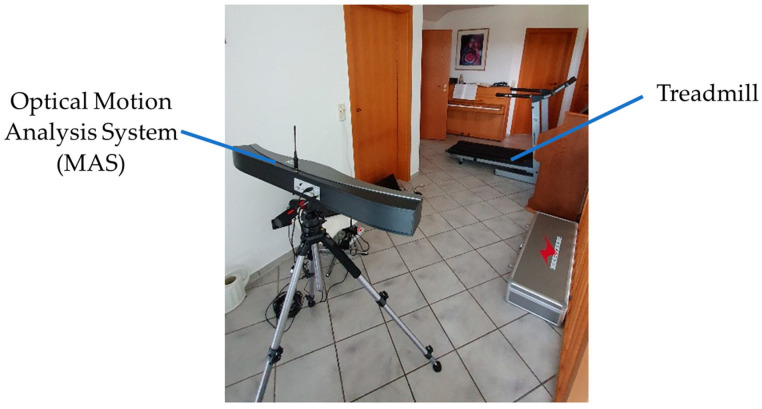
Treadmill test setup with optical MAS positioned 3 m away from the treadmill.

With measurements on a treadmill, the behavior of the sensor accuracy under real conditions when walking at different velocities is tested. The goal is to compare the angle progression measured by SensAA with that of MAS. The setup of the treadmill test is shown in Figure 8.

The wearable Sensor Boxes are attached to a healthy subject’s thigh and shank of the right leg with elastic bandages. These are easy to put on and off and are adjustable to the thickness of the wearer’s leg.

**Figure 9:**
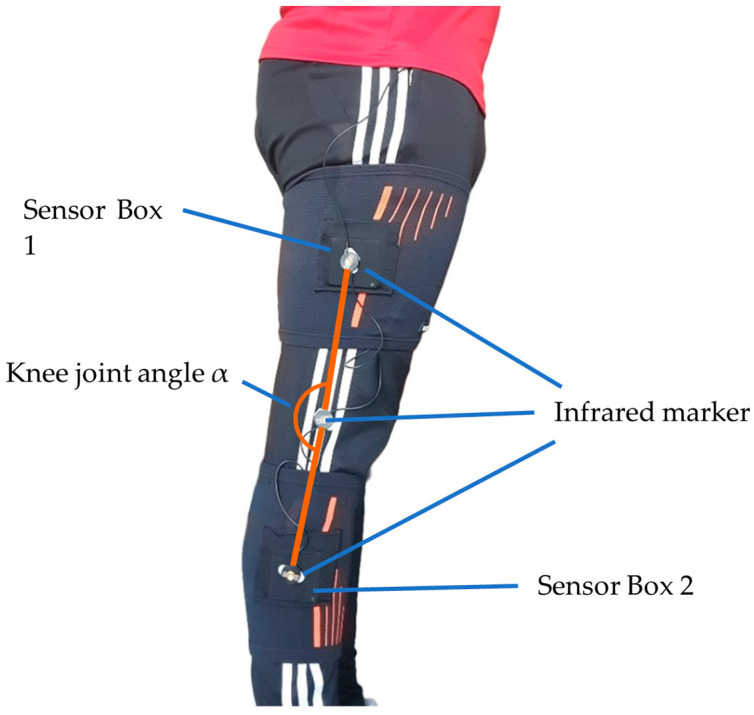
Sensor Boxes attached with bandages to the thigh and shank of the right leg. Additionally, three infrared markers for an optical MAS are attached to the leg. They form a triangle that covers the angle α of the knee joint.

As shown in Figure 9, the infrared markers of the MAS are mounted centered on the Sensor Boxes. An additional marker is located at the rotation point of the knee joint to form a triangle with the markers on the thigh and the shank when the knee is flexed. From this triangle, the knee angle is calculated.

To perform the test, both SensAA and the MAS are started, and the treadmill is initially set to 3 km/h walking speed. A dataset over at least 1 min is recorded before the speed is gradually increased. The experiment was carried out three times at walking speeds of 3 km/h, 6 km/h, 9 km/h and 12 km/h. When the speed is increased to 12 km/h, the subject must change their gait from walking to jogging. This test serves as a system load test to evaluate how the quality of the knee angle progression performs at high speed.

The calculated data are transmitted via BLE and stored in the Cloud Server System as described in Section 2.1, Section 2.3 and Section 2.4.

## 3. Results

### 3.1. Battery Life Test

**Figure 10:**
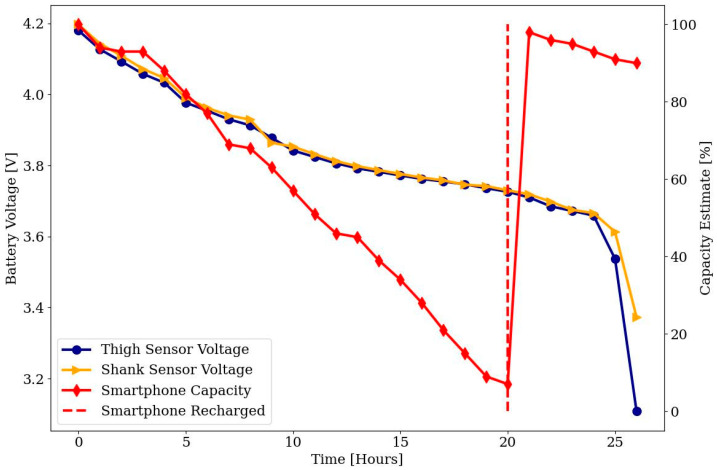
Discharge curves of the batteries of the sensor modules at the shank and thigh and the smartphone.

The discharge curves of the Sensor Boxes’ batteries and the capacity estimation of the smartphone during the battery life test are shown in Figure 10 (see Section 2.5.1). After approx. 26 h of runtime, the batteries dropped below the 3.3 V threshold, at which the sensor module can no longer be supplied, and the data recording stops. The discharge curves of the batteries showed typical Li-Ion behavior. The smartphone’s battery level was at 7% after 20 h. The battery was then recharged, but the data recording continued.

### 3.2. Pendulum Test

**Figure 11:**
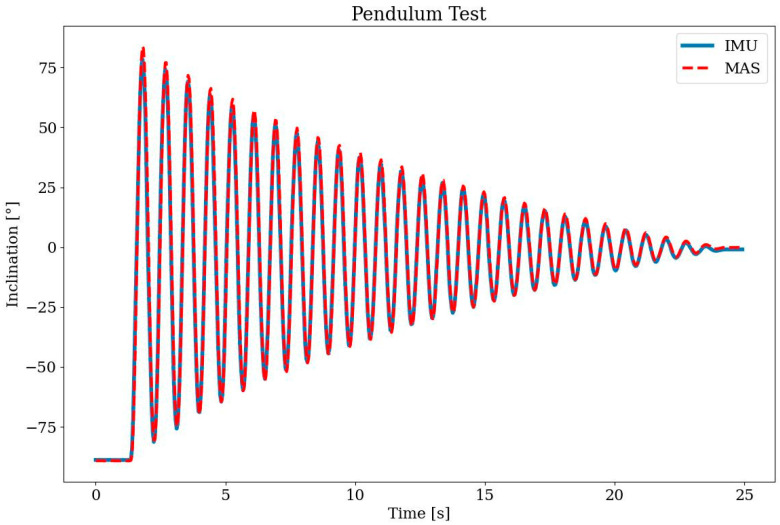
Results of the pendulum test; knee joint angle of the IMU sensors of SensAA in comparison to reference MAS.

The result of the pendulum test (see Section 2.5.2) is shown in Figure 11. The graph shows the comparison of the measured angle obtained from the MAS and the proposed measurement system. The main metric used to quantify the comparison was the Euclidean distance between the two datasets, also referred to as the Root Mean Square Error (RMSE). To further evaluate the quality of the measurements, the difference of the angle curves and their mean value were calculated. For this purpose, the mean standard deviation was used, which reflects the average distance of all measured points from the mean value. Table 1 summarizes the results.

### 3.3. Treadmill Test

**Figure 12:**
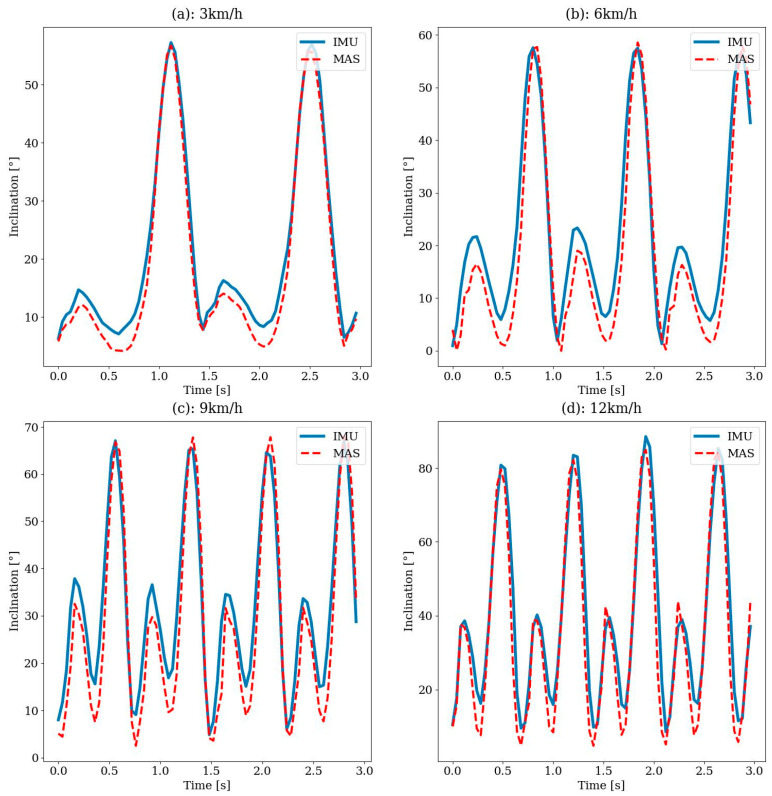
Comparison of the knee angle measured by IMU sensors of SensAA and reference MAS on treadmill at (**a**) 3 km/h, (**b**) 6 km/h, (**c**) 9 km/h and (**d**) 12 km/h walking speed.

To verify the wearable sensor data, the resulting knee angle signal was compared to the results of the MAS system. First, the offset in the time axis of both signals was determined and applied to the data. Then, both datasets were interpolated onto the same sampling time points. Since the MAS system has a significantly higher sampling rate compared to SensAA, the time points of the wearable sensor data were used for interpolation. Figure 12 shows both signals compared to each other. To quantify the comparison between the two datasets, the RMSE of SensAA in reference to the MAS was calculated. Additionally, the average, minimum, and maximum distance and the standard deviation between both signals were determined. Table 2 shows the comparison results. The lowest RMSE value was measured for a speed of 3 km/h at 2.9°. For speeds of 6 and 9 km/h, the RMSE was at 6.1° and 5.2°. The highest RMSE of 8.0° was reached at 12 km/h. The average deviation was below 5° at all four walking speeds, and the standard deviation was between 1.6° and 6.7°.

**Figure 13:**
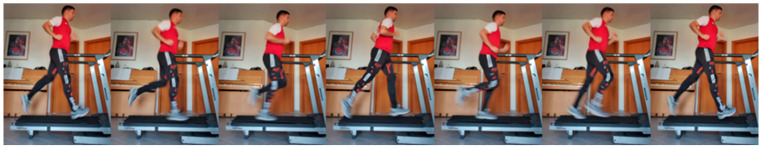
Gait cycle on the treadmill at 12 km/h.

Figure 13 shows the phases of the gait cycle at a speed of 12 km/h. At such speeds, there were phases in which both feet no longer touched the ground, and as a result, the test person bounced off the ground. In the fifth sequence, it can be recognized that the knee angle experienced a significantly greater maximum in the swing phase compared to walking. Here, the maximum knee angle was between 80° and 90°, which can be seen from the knee angle curve in Figure 12. For comparison, the maximum knee angle ranged between 60° and 70° during walking.

## 4. Discussion

In this paper, SensAA is proposed, a low-cost, highly mobile biomechanical measurement system capable of measuring the angle of the knee joint based on inertial measurement units. The system uses centralized cloud-hosted data storage and an Android smartphone to design a solution that is both inexpensive (see Section 2.5) and easy to set up. With BLE as the wireless transmission protocol, a battery life of more than 24 h was achieved. This result is well suited for long-term recordings. It should be mentioned that no other activities were performed on the smartphone during the test. No extensive screen time or other activities should be carried out on the smartphone during long-term recording.

The proposed system was first verified with a pendulum test. For angle measurement accuracy, an RMSE of 3.5° and standard deviation of 3.4° were achieved. This proves that the implemented observer algorithm can calculate the angle between two Sensor Boxes in the sagittal plane accurately.

The system was further tested with a treadmill test at different walking speeds. This test had the purpose of a first-time technical verification of the system on a human body. For this reason, only one participant took part in the measurements. The acquired results were dependent on the individual subject characteristics (e.g., age, gender, height) and did not give a reliable answer regarding the overall accuracy of the system. To acquire this information, a higher number of participants is needed in the future. However, the presented results show that SensAA works in general and has the potential to reach one of the highest overall accuracies compared to other systems.

SensAA achieved an RMSE of 2.9° to 8° for walking speeds between 3 and 12 km/h. The results of walking at 3 km/h deviated less than those of most previous studies, which reported RMSE values between 3.2° and 5.9° [25,26,27,28,33,34,37,41]. These studies describe the walking activity during their measurement routine only as walking and do not give an exact speed. Therefore, no comparison of the measuring accuracy of the systems at the exact same speed can be made.

To our knowledge, only Robert-Lachaine et al. analyzed the motion of the human body at specified speeds [36]. They investigated speeds from 0.6 to 1.2 m/s. At a speed of 0.8 m/s, which is about 3 km/h, the RMSE was 3.2°. Compared to this result, SensAA achieved a smaller RMSE at the same walking speed.

Joukov et al. [32] and Teufl et al. [40] achieved lower errors, with RMSE values of 2.5° and 2.38° to 2.65°. Joukov et al. [32] proposed a system based on a rhythmic extended Kalman filter, which considers the periodicity of movements when walking. This significantly improves the state estimation of a regular EKF algorithm. Teufl et al. [40] achieved high accuracy by following an iterated extended Kalman filter approach. The inertial data were processed twice, first to obtain a converged estimate of the acceleration bias that was then used as an initial guess for the second calculation [40]. Compared to SensAA, the downside of these two algorithms is the increased computing effort and runtime.

The RMSE values of SensAA increased with a higher walking speed. Other studies have experienced the same problem with increasing complexity of speed and increasing gait movement [27,33]. It should be noted that the error for the speed of 6 km/h (RMSE 6.1°) was greater than for 9 km/h (RMSE 5.2°), which was contrary to the expected trend. One explanation could be a slightly loose attachment of one Sensor Box during the 6 km/h run, which caused false sensor inclination calculations.

The higher increase in error compared to other studies might be caused by the way the Sensor Boxes are mounted. Sensors were not attached directly to the skin but to the participant’s pants. Fast walking leads to movement of the fabric, which could again result in inclination of the sensor and false calculations.

In order to achieve high accuracy for the measurement of joint angles using IMU sensors, Niswander et al. [54] point out that the positioning of the sensors must be optimized. They compared different sensor positions and reached the lowest RMSE for knee angle with sensors positioned at the middle lateral shank and the lower anterior thigh [54]. In addition to these sensor positions, they also recommended the positioning that has been chosen for SensAA.

Another crucial aspect of MAS is the sensor calibration procedure [27,29,55]. Di Raimondo et al. [27] describe three possible types of IMU calibration: manual, static, and functional calibration. They propose a functional calibration method that integrates hip abduction-adduction motion, sit-to-stand, and walking movements. With this method, the RMSE could be reduced from 7.8°, with manual calibration, to 3.9°. SensAA is calibrated with a static method.

It must be considered that SensAA cannot be compared to a reliable ground truth. The optical reference system is also subject to errors. Teufl et al. [40] showed that MAS based on optical markers can suffer from soft tissue artefacts (STA) when the markers are placed on anatomical landmarks (cp. [30,33,37]). Teufl et al. [40] achieved higher accuracy by using rigid marker clusters fixed on the thigh and shank with straps (RMSE reduction of up to 1.3°). For the treadmill test, we attached two markers to the rigid Sensor Boxes, but one marker on the rotation axis of the knee. This marker might be affected by STA in addition to the fabric movement.

The measuring accuracy of SensAA could possibly be improved by optimizing the sensor attachment, sensor positions, and the calibration method. However, SensAA shows viable performance for gait analysis purposes. Errors between 2° and 5° are considered clinically acceptable [27]. This threshold was achieved by walking at 3 km/h. Speeds from 6 km/h to 12 km/h are rarely measured in everyday life situations, especially for elderly people [56,57].

At this point, SensAA has been tested in a laboratory. However, the tests conducted showed that the proposed toolchain of joint angle measuring and cloud-based data storage works as desired. The system produces clinically acceptable results for knee joint angle measurements and is applicable for long-term observation in everyday life.

## 5. Conclusions and Outlook

In this paper, we introduced SensAA, a wearable cloud-based biomechanical data acquisition system. The system is verified for long-term measurements of the human knee joint angle. It uses low-cost commercially available components and open-source software. SensAA is an all-in-one solution that provides high accuracy, high usability, and high security.

While SensAA demonstrates promising performance, further optimizations are necessary to ensure consistent accuracy for walking speeds exceeding 3 km/h. Future plans include conducting a long-term study with human test subjects to validate the system’s performance in real-world environments.

For future work, SensAA can be used to calculate realistic load collectives of the human leg and identify different walking activities. These measurements could be used for designing and controlling an active exoskeleton, which assists the user with ADLs like walking, climbing stairs, sitting down, and standing up.

The system is not limited to the knee joint and the lower limbs but can be used on the whole body. Additional Sensor Boxes could be used to examine other human joints simultaneously. SensAA can also be used not only for measuring joint angles, but also for measuring the acceleration and angular velocity of body parts.

SensAA provides a powerful, scalable platform for collecting and processing biomechanical data. Thanks to the cloud-based data storage solution, examinations of a large group of participants can be highly automated. This could help create an extensive database for human kinematics.

## Figures and Tables

**Table 1:** Pendulum test results, angle difference between SensAA and reference MAS.

RMSE	Standard Deviation	Average Difference
3.5°	3.4°	2.3°

**Table 2:** Verification results of the treadmill tests, from 3 km/h up to 12 km/h.

Walking Speed	RMSE	Standard Deviation	Difference		
			Average	Minimum	Maximum
3 km/h	2.9°	1.6°	2.4°	−3.0°	7.0°
6 km/h	6.1°	5.1°	3.4°	−11.1°	18.2°
9 km/h	5.2°	5.1°	1,0°	−10.1°	16.3°
12 km/h	8.0°	6.7°	4.3°	−11.9°	24.2°

## Data Availability

The data presented in this study are available on request from the corresponding author.

## Abbreviations

The following abbreviations are used in this manuscript:

ADL	Activities of Daily Living
API	Application Programming Interface
BLE	Bluetooth^®^ Low Energy
ECG	Electrocardiography
EIT	Electrical Impedance Tomography
EKF	Extended Kalman Filter
EMG	Electromyography
GATT	Generic Attribute Profile
GRF	Ground Reaction Force
HTTP	Hypertext Transfer Protocol
HTTPS	Hypertext Transfer Protocol Secure
IMU	Inertial Measurement Unit
IP	Internet Protocol
JSON	Java Script Object Notation
LDO	Low Drop-Out Voltage Regulator
LiPo	Lithium Polymer
LTE	Long Term Evolution
MAS	Motion Analysis System
MEMS	Micro-Electro-Mechanical Systems
OLED	Organic Light Emitting Diode
OPD	Organic Photo Diode
PCB	Printed Circuit Board
RMS	Root Mean Squared
RMSE	Root Mean Squared Error
STA	Soft Tissue Artifacts
SQL	Structured Query Language
TLS	Transport Layer Security
USB	Universal Serial Bus

## References

[1] Pirker W., Katzenschlager R. (2017). Gait disorders in adults and the elderly: A clinical guide. Wien. Klin. Wochenschr..

[2] Mahlknecht P., Kiechl S., Bloem B.R., Willeit J., Scherfler C., Gasperi A., Rungger G., Poewe W., Seppi K. (2013). Prevalence and burden of gait disorders in elderly men and women aged 60–97 years: A population-based study. PLoS ONE.

[3] Meda-Gutiérrez J.R., Zúñiga-Avilés L.A., Vilchis-González A.H., Ávila-Vilchis J.C. (2021). Knee Exoskeletons Design Approaches to Boost Strength Capability: A Review. Appl. Sci..

[4] Nesler C., Thomas G., Divekar N., Rouse E.J., Gregg R.D. (2022). Enhancing Voluntary Motion with Modular, Backdrivable, Powered Hip and Knee Orthoses. IEEE Robot. Autom. Lett..

[5] Toxiri S., Calanca A., Poliero T., Caldwell D.G., Ortiz J., Carrozza M.C., Micera S., Pons J.L., Carrozza M.C. (2019). Actuation Requirements for Assistive Exoskeletons: Exploiting Knowledge of Task Dynamics. Wearable Robotics: Challenges and Trends, Proceedings of the 4th International Symposium on Wearable Robotics, WeRob2018, Pisa, Italy, 16–20 October 2018.

[6] Carrozza M.C., Micera S., Pons J.L., Carrozza M.C. (2019). Wearable Robotics: Challenges and Trends, Proceedings of the 4th International Symposium on Wearable Robotics, WeRob2018, Pisa, Italy, 16–20 October 2018.

[7] Arazpour M., Bani M.A., Hutchins S.W. (2013). Reciprocal gait orthoses and powered gait orthoses for walking by spinal cord injury patients. Prosthet. Orthot. Int..

[8] Contreras-Vidal J.L., Bhagat A.N., Brantley J., Cruz-Garza J.G., He Y., Manley Q., Nakagome S., Nathan K., Tan S.H., Zhu F. (2016). Powered exoskeletons for bipedal locomotion after spinal cord injury. J. Neural Eng..

[9] Alqahtani M.S., Al-Tamimi A., Almeida H., Cooper G., Bartolo P. (2020). A review on the use of additive manufacturing to produce lower limb orthoses. Prog. Addit. Manuf..

[10] Tarbit J., Hartley N., Previte J. (2023). Exoskeletons at your service: A multi-disciplinary structured literature review. J. Serv. Mark..

[11] Yan T., Cempini M., Oddo C.M., Vitiello N. (2015). Review of assistive strategies in powered lower-limb orthoses and exoskeletons. Robot. Auton. Syst..

[12] Willwacher S., Robbin J., Eßer T., Mai P. (2023). Bewegungsanalysesysteme in der Forschung und für niedergelassene Orthopädinnen und Orthopäden. Orthopadie.

[13] Riener R., Rabuffetti M., Frigo C. (2002). Stair ascent and descent at different inclinations. Gait Posture.

[14] Laudanski A., Brouwer B., Li Q. (2013). Measurement of lower limb joint kinematics using inertial sensors during stair ascent and descent in healthy older adults and stroke survivors. J. Healthc. Eng..

[15] Brinckmann P. (2012). Orthopädische Biomechanik: Mit 23 Tabellen; Mit Einem Verzeichnis der Fachausdrücke der Orthopädischen Biomechanik Englisch-Deutsch.

[16] Jasiewicz J.M., Allum J.H.J., Middleton J.W., Barriskill A., Condie P., Purcell B., Li R.C.T. (2006). Gait event detection using linear accelerometers or angular velocity transducers in able-bodied and spinal-cord injured individuals. Gait Posture.

[17] Gao F., Liu G., Liang F., Liao W.-H. (2020). IMU-Based Locomotion Mode Identification for Transtibial Prostheses, Orthoses, and Exoskeletons. IEEE Trans. Neural Syst. Rehabil. Eng..

[18] Wong Z.Y., Ishak A.J., Ahmad S.A., Chong Y.Z. (2014). Mechanical analysis of wearable lower limb exo-skeleton for rehabilitation. J. Eng. Sci. Technol..

[19] Zhang Y., Ma Z., Zuo S., Liu J. Gait Prediction and Assist Control of Lower Limb Exoskeleton Based on Inertia Measurement Unit. Proceedings of the 2022 5th International Conference on Intelligent Robotics and Control Engineering (IRCE).

[20] Gonzales-Huisa O.A., Oshiro G., Abarca V.E., Chavez-Echajaya J.G., Elias D.A. (2023). EMG and IMU Data Fusion for Locomotion Mode Classification in Transtibial Amputees. Prosthesis.

[21] Stetter B.J., Krafft F.C., Ringhof S., Stein T., Sell S. (2020). A Machine Learning and Wearable Sensor Based Approach to Estimate External Knee Flexion and Adduction Moments during Various Locomotion Tasks. Front. Bioeng. Biotechnol..

[22] Zihajehzadeh S., Park E.J. (2017). A Novel Biomechanical Model-Aided IMU/UWB Fusion for Magnetometer-Free Lower Body Motion Capture. IEEE Trans. Syst. Man Cybern, Syst..

[23] Hamdi Mohammed M., Awad Mohammed I., Abdelhameed Magdy M., Tolbah Farid A. Lower limb motion tracking using IMU sensor network. Proceedings of the 2014 Cairo International Biomedical Engineering Conference (CIBEC).

[24] Iman P., Doik K. Design and implementation of IMU-based human arm motion capture system. Proceedings of the 2012 IEEE International Conference on Mechatronics and Automation.

[25] Carcreff L., Payen G., Grouvel G., Massé F., Armand S. (2022). Three-Dimensional Lower-Limb Kinematics from Accelerometers and Gyroscopes with Simple and Minimal Functional Calibration Tasks: Validation on Asymptomatic Participants. Sensors.

[26] Cerfoglio S., Capodaglio P., Rossi P., Conforti I., D’Angeli V., Milani E., Galli M., Cimolin V. (2023). Evaluation of Upper Body and Lower Limbs Kinematics through an IMU-Based Medical System: A Comparative Study with the Optoelectronic System. Sensors.

[27] Di Raimondo G., Vanwanseele B., van der Have A., Emmerzaal J., Willems M., Killen B.A., Jonkers I. (2022). Inertial Sensor-to-Segment Calibration for Accurate 3D Joint Angle Calculation for Use in OpenSim. Sensors.

[28] Dorschky E., Nitschke M., Seifer A.-K., van den Bogert A.J., Eskofier B.M. (2019). Estimation of gait kinematics and kinetics from inertial sensor data using optimal control of musculoskeletal models. J. Biomech..

[29] Favre J., Aissaoui R., Jolles B.M., Guise J.A.d., Aminian K. (2009). Functional calibration procedure for 3D knee joint angle description using inertial sensors. J. Biomech..

[30] Finco M.G., Patterson R.M., Moudy S.C. (2023). A pilot case series for concurrent validation of inertial measurement units to motion capture in individuals who use unilateral lower-limb prostheses. J. Rehabil. Assist. Technol. Eng..

[31] Gard S.A.P. (2006). Use of Quantitative Gait Analysis for the Evaluation of Prosthetic Walking Performance. JPO J. Prosthet. Orthot..

[32] Joukov V., Bonnet V., Karg M., Venture G., Kulic D. (2018). Rhythmic Extended Kalman Filter for Gait Rehabilitation Motion Estimation and Segmentation. IEEE Trans. Neural Syst. Rehabil. Eng..

[33] Lebleu J., Gosseye T., Detrembleur C., Mahaudens P., Cartiaux O., Penta M. (2020). Lower Limb Kinematics Using Inertial Sensors during Locomotion: Accuracy and Reproducibility of Joint Angle Calculations with Different Sensor-to-Segment Calibrations. Sensors.

[34] Lora-Millan J.S., Hidalgo A.F., Rocon E. (2021). An IMUs-Based Extended Kalman Filter to Estimate Gait Lower Limb Sagittal Kinematics for the Control of Wearable Robotic Devices. IEEE Access.

[35] Poitras I., Dupuis F., Bielmann M., Campeau-Lecours A., Mercier C., Bouyer L.J., Roy J.-S. (2019). Validity and Reliability of Wearable Sensors for Joint Angle Estimation: A Systematic Review. Sensors.

[36] Robert-Lachaine X., Parent G., Fuentes A., Hagemeister N., Aissaoui R. (2020). Inertial motion capture validation of 3D knee kinematics at various gait speed on the treadmill with a double-pose calibration. Gait Posture.

[37] Seel T., Raisch J., Schauer T. (2014). IMU-based joint angle measurement for gait analysis. Sensors.

[38] Shull P.B., Jirattigalachote W., Hunt M.A., Cutkosky M.R., Delp S.L. (2014). Quantified self and human movement: A review on the clinical impact of wearable sensing and feedback for gait analysis and intervention. Gait Posture.

[39] Storm F.A., Cesareo A., Reni G., Biffi E. (2020). Wearable Inertial Sensors to Assess Gait during the 6-Minute Walk Test: A Systematic Review. Sensors.

[40] Teufl W., Miezal M., Taetz B., Fröhlich M., Bleser G. (2018). Validity, Test-Retest Reliability and Long-Term Stability of Magnetometer Free Inertial Sensor Based 3D Joint Kinematics. Sensors.

[41] Weygers I., Kok M., Konings M., Hallez H., Vroey H.d., Claeys K. (2020). Inertial Sensor-Based Lower Limb Joint Kinematics: A Methodological Systematic Review. Sensors.

[42] Cloete T., Scheffer C. Benchmarking of a full-body inertial motion capture system for clinical gait analysis. Proceedings of the 2008 30th Annual International Conference of the IEEE Engineering in Medicine and Biology Society.

[43] Robert-Lachaine X., Mecheri H., Muller C., Larue C., Plamondon A. (2020). Validation of a low-cost inertial motion capture system for whole-body motion analysis. J. Biomech..

[44] Benson L.C., Räisänen A.M., Clermont C.A., Ferber R. (2022). Is This the Real Life, or Is This Just Laboratory? A Scoping Review of IMU-Based Running Gait Analysis. Sensors.

[45] O’Reilly M., Caulfield B., Ward T., Johnston W., Doherty C. (2018). Wearable Inertial Sensor Systems for Lower Limb Exercise Detection and Evaluation: A Systematic Review. Sports Med..

[46] Robert-Lachaine X., Mecheri H., Larue C., Plamondon A. (2017). Validation of inertial measurement units with an optoelectronic system for whole-body motion analysis. Med. Biol. Eng. Comput..

[47] Amin J., Ruthiraphong P. Cloud-based Gait Analysis Using a Single IMU for Parkinson Disease. Proceedings of the 2021 18th International Conference on Electrical Engineering/Electronics, Computer, Telecommunications and Information Technology (ECTI-CON).

[48] Al Borno M., O’Day J., Ibarra V., Dunne J., Seth A., Habib A., Ong C., Hicks J., Uhlrich S., Delp S. (2022). OpenSense: An open-source toolbox for inertial-measurement-unit-based measurement of lower extremity kinematics over long durations. J. Neuroeng. Rehabil..

[49] ST Microelectronics STEVAL-STLCS02V1—Data Brief. https://www.st.com/resource/en/data_brief/steval-stlcs02v1.pdf.

[50] ST Microelectronics LSM6DSM—iNEMO Inertial Module: Always-On 3D Accelerometer and 3D Gyroscope. https://www.st.com/resource/en/datasheet/lsm6dsm.pdf.

[51] Adafruit Industries Adafruit Micro-Lipo Charger for LiPo/LiIon Batt w/MicroUSB Jack. https://www.adafruit.com/product/1904.

[52] Pachi A., Ji T. (2005). Frequency and Velocity of People Walking.

[53] Karampour H., Piran F., Faircloth A., Talebian N., Miller D. (2023). Vibration of Timber and Hybrid Floors: A Review of Methods of Measurement, Analysis, and Design. Buildings.

[54] Niswander W., Wang W., Kontson K. (2020). Optimization of IMU Sensor Placement for the Measurement of Lower Limb Joint Kinematics. Sensors.

[55] Pacher L., Chatellier C., Vauzelle R., Fradet L. (2020). Comparison of lower limb calibration methods for movement analysis with inertial measurement unit (IMU). Comput. Methods Biomech. Biomed. Eng..

[56] Prakash C., Kumar R., Mittal N. (2018). Recent developments in human gait research: Parameters, approaches, applications, machine learning techniques, datasets and challenges. Artif. Intell. Rev..

[57] Kramers-De Quervain I.A., Stüssi E., Stacoff A. (2008). Ganganalyse beim Gehen und Laufen. Schweiz. Z. Sportmed. Sport.

